# High resistance of *Rhipicephalus microplus* to acaricides in the Recôncavo baiano, state of Bahia, Brazil

**DOI:** 10.1590/S1984-29612026028

**Published:** 2026-07-20

**Authors:** Juan Dario Puentes, Wendell Marcelo de Souza Perinotto, Ana Maria dos Santos Lima, Larissa Claudino Ferreira, Ana Caroline Dantas Amorim, Vinícius Longo Ribeiro Vilela, Franklin Riet-Correa

**Affiliations:** 1 Universidade Federal da Bahia - UFBA, Programa de Pós-graduação em Ciência Animal nos Trópicos - PPGCAT, Salvador, BA, Brasil; 2 Universidade Federal Rural do Rio de Janeiro - UFRRJ, Programa de Pós-graduação em Ciências Veterinárias - PPGCV, Seropédica, RJ, Brasil; 3 Instituto Federal da Paraíba - IFPB, Departamento de Medicina Veterinária, Sousa, PB, Brasil; 4 Universidade Federal de Campina Grande - UFCG, Programa de Pós-Graduação em Ciência e Saúde Animal, Patos, PB, Brasil

**Keywords:** Acaricide resistance, cattle farms, cattle tick, larval bioassays, multiple resistance, Resistência a acaricidas, propriedades pecuárias, carrapato-do-boi, bioensaios larvais, resistência múltipla

## Abstract

This research aimed to quantify resistance levels to cypermethrin, chlorpyrifos, amitraz, ivermectin, and fipronil in *Rhipicephalus microplus* larvae from cattle farms in the Recôncavo baiano region of the State of Bahia, Brazil. We also identified associations between resistance level and operational factors. Engorged adult female ticks were collected from 14 farms to perform bioassays for the five acaricide classes and determine the intensity of resistance. A nonparametric Mann-Whitney U test was employed to identify categorical factors associated with resistance intensities. A high frequency of resistance was observed across all five compounds evaluated, even on farms that reported never using these products. On all farms, we found tick populations resistant to at least three acaricides. While cypermethrin, chlorpyrifos, and ivermectin showed the highest intensity of resistance (RR_50_: 38.06, 126, and 27.35, respectively), amitraz and fipronil showed the lowest (RR_50_: 11.28 and 1.06, respectively). In the Recôncavo baiano region, the incorporation of amitraz and fipronil in the formulation of strategic control protocols is recommended.

## Resumo

Esta pesquisa teve como objetivo quantificar o nível de resistência à cipermetrina, clorpirifós, amitraz, ivermectina e fipronil em larvas de *Rhipicephalus microplus* provenientes de fazendas bovinas da região do Recôncavo baiano do estado da Bahia, Brasil. Identificamos também associações entre o nível de resistência e fatores de manejo. Foram coletadas fêmeas adultas ingurgitadas em 14 propriedades para determinação da intensidade da resistência aos cinco acaricidas por meio de bioensaios. Realizou-se o teste não paramétrico de Mann-Whitney para identificar fatores associados à intensidade de resistência. Foi observada uma alta frequência de resistência aos cinco compostos avaliados, inclusive em fazendas que relataram nunca ter utilizado amitraz ou fipronil. Populações de carrapatos resistentes a pelo menos três acaricidas foram encontradas em todas as propriedades avaliadas. Enquanto cipermetrina, clorpirifós e ivermectina apresentaram os maiores níveis de resistência (RR_50_: 38,06, 126, e 27,35, respectivamente), amitraz e fipronil apresentaram os menores (RR_50_: 11,28 e 1,06, respectivamente). Recomenda-se o uso de amitraz e fipronil na formulação de protocolos de controle estratégico na região do Recôncavo baiano.

## Introduction

Cattle tick, *Rhipicephalus* (*Boophilus*) *microplus,* is an ectoparasite that affects cattle and causes high losses for Brazilian livestock (Grisi et al., 2014). Acaricides are chemical compounds largely used for cattle tick control. The most used chemical groups (active ingredients) are synthetic pyrethroids (SPs) (e.g., cypermethrin), organophosphates (OPs) (e.g., chlorpyrifos), formamidines (e.g., amitraz), macrocyclic lactones (MLs) (e.g., ivermectin), phenilpyrazoles (e.g., fipronil), isoxazolines (e.g., fluralaner), and benzoylphenylureas (e.g., fluazuron) (Rojas-Cabeza et al., 2025). These acaricides act on the tick by targeting different sites: voltage-gated sodium channels; acetylcholinesterase inhibitors; octopamine and tyramine receptors; glutamate-gated chloride channels; gamma-aminobutyric acid - gated chloride channels; and chitin synthase, respectively (Obaid et al., 2022; FAO, 2025).

Acaricide resistance is a genetic phenomenon in which some individuals are selected over time to survive exposure to acaricides compared to most individuals of the species (at least at the time of the drug’s release to the market) or to a reference susceptible strain. This ability increases with time in the population (Klafke et al., 2024). The current literature classifies factors that hasten acaricide resistance selection into genetic, biological, and operational. The genetic factors include dominance of resistance alleles, number of genes involved, initial frequency of resistance genes, genetic diversity of population, relative fitness of resistant organisms, chance of linkage disequilibrium, and opportunity for genetic recombination; however, those factors are hard to manage as they are not under human control (Chevillon et al., 2013). Biological factors comprise biotic and behavioral aspects of ticks. Operational factors include the chemical nature of the drug, the possibility of cross-resistance, drug persistence in the host, drug clearance kinetics, and the frequent use of the same acaricide for prolonged periods (Abbas et al., 2014).

In Brazil, it has been documented acaricide resistance for almost all compounds for several years. In the State of Rio Grande do Sul, the first report on OPs’ resistance was published in 1972 and the first report of SPs’ resistance, in 1989. In the State of Rio de Janeiro, the first case of amitraz resistance was published in 1993 (Klafke et al., 2024). In the State of Rio Grande do Sul, Martins and Furlong conducted the first report of MLs’ resistance in 2001 (Martins & Furlong, 2001). In the States of Minas Gerais, Rio Grande do Sul, and São Paulo, Castro-Janer et al. performed the first report of fipronil resistance in 2010 (Castro-Janer et al., 2010). In the State of Rio Grande do Sul, Reck et al. reported the first case of fluazuron resistance in 2014 (Reck et al., 2014). After the first report of resistance for each acaricide class, numerous studies have documented additional cases of resistance. Until now, the isoxazoline group (e.g., fluralaner) remains the only class with no resistance documented.

Diagnosis of acaricidal resistance is the first step for rational tick control, and larval tests are the most sensitive approach for detecting resistant phenotypes in a tick strain. The larval tests allow for the assessment of resistance levels and are frequently used in epidemiological studies (Klafke et al., 2024). Despite existing reports of acaricide resistance in the State of Bahia, there is a lack of recent studies using more accurate diagnostic techniques (Campos Júnior & Oliveira, 2005; Spagnol et al., 2010; Raynal et al., 2018). In this research, we aimed to quantify resistance levels to cypermethrin, chlorpyrifos, amitraz, ivermectin, and fipronil in *R. microplus* larvae from cattle farms in the Recôncavo baiano region of the State of Bahia, Brazil. We also explored associations between the resistance level and operational factors.

## Material and Methods

### Study localization

**Figure 1:**
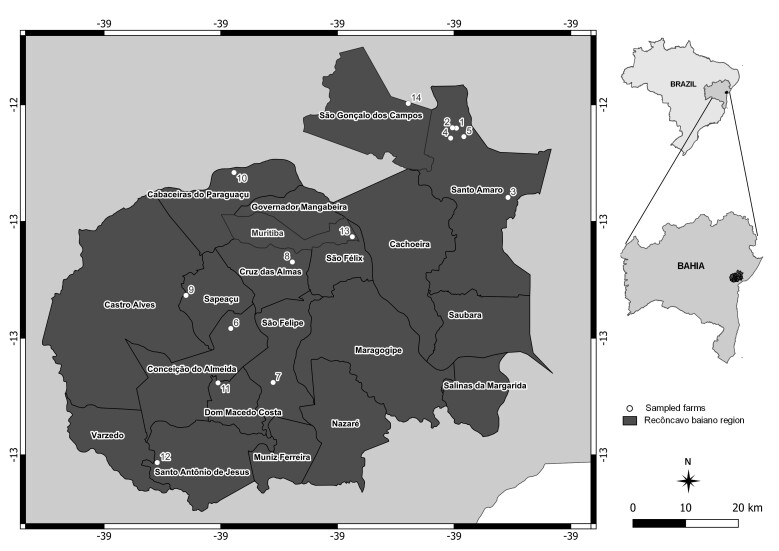
Localization of the sampled farms. The map shows the municipalities that integrate the Recôncavo baiano region. The dark area indicates the extent of the territory, and the white points indicate the locations of the 14 sampled farms.

We conducted this research in the Recôncavo baiano region, in the state of Bahia, Brazil. The region lies within the Atlantic Forest biome and, according to the Köppen-Geiger climate classification, has a humid tropical climate (Af), characterized by two well-defined seasons. The summer, from October to March, is rainy, with accumulated precipitation of 481.7 mm, an average temperature of 26.65°C (±2.29), and a relative humidity of 90.93% (±7.13). The winter, from April to September, is also rainy, with accumulated precipitation of 479.4 mm, an average temperature of 24,88°C (±2.08), and a relative humidity of 96,04% (±4.98) (Alvares et al., 2013). Ticks of the species *R. microplus* were sampled in 14 farms from the municipalities of Santo Amaro da Purificação, Conceição do Almeida, São Felipe, Cruz das Almas, Sapeaçú, Cabaceiras do Paraguaçú, Dom Macedo Costa, Santo Antônio de Jesus, Muritiba, and São Gonçalo dos Campos (Figure 1).

### Experimental design

A cross-sectional analytical study was designed to evaluate the degree of resistance to acaricide compounds on farms in the Recôncavo baiano region, State of Bahia. Between 2022 and 2024, we sampled cattle tick populations from 14 farms selected by convenience sampling. The selection criterion for the farms included in the study was the absence of tick treatment for at least 45 days prior to sampling to avoid interference with the bioassays. All farmers provided consent after being informed about the sampling objectives. The procedures were approved by the local committee on animal experimentation (CEUA-UFRB Nº 55/2023).

### Questionnaire

Information was obtained from each farm owner or manager to characterize the farms and to identify factors associated with resistance to acaricide compounds. Researchers interviewed each person individually and explained the risks and benefits of the research. Data obtained from 13 questions included information on farm characteristics: herd size; genetic composition (*Bos indicus* or *Bos taurus* and crossbred cattle); production system (beef or dairy); and region from which the purchased cattle came (from the Caatinga biome or from the Atlantic Forest biome). Also included details about tick control practices: tick infestation seasonality (all through the year or part of the year); acaricide application criteria (systematically or only when cattle was highly infested); acaricide purchase criteria (personal election or veterinary recommendation without resistance test); treatment frequency (≤2 or >2 treatments by year); formulation of acaricide used (injectable, pour-on or spray formulations); and management acaricide practices (rotation of acaricide compound, use of multiple acaricides classes simultaneously, use of different products with same compound). All farmers provided consent after being informed about the questionnaire objectives. The interview was approved by the Research Ethics Committee (CEP-UFRB No. 6.586.232/2023).

### Preparation of ticks

The Porto Alegre (POA) isolate was used as a reference susceptible strain of *R. microplus*. Since its initial isolation, the POA strain has been widely used as a susceptible reference strain (Reck et al., 2014; Vilela et al., 2020; Ferreira et al., 2023).

The sample conditioning was performed according to the recommendations of the Food and Agriculture Organization of the United Nations (FAO, 2025). A sample of 10 to 50 engorged female adult *R. microplus* ticks was collected from each farm. Infested cattle were restrained using cattle squeeze chute or ropes, and ticks were manually removed. Each tick population was deposited in perforated plastic jars and transported immediately to the laboratory. Upon arrival, the ticks were washed with distilled water, dried with paper towels, and placed in Petri dishes (ten ticks per dish). The dishes were incubated in a Biological Oxygen Demand (BOD) chamber at 27 ± 1 °C and 80 ± 5% relative humidity. After 14 days, when oviposition ended, the eggs were recovered, homogenized, and 500mg were placed in a 10mL plastic vial, which was capped with a cotton plug. Five flasks of each farm were transported to the Laboratório de Parasitologia Veterinária (LPV) of the Instituto Federal da Paraíba, Sousa, Paraíba, Brazil, where they were incubated under the same conditions of the oviposition until larval hatching ended.

### Acaricide resistance bioassays

To determine the intensity of resistance, bioassays were performed for the five acaricide classes that best represented their pharmacologic families. The chemical compounds employed in the evaluation were technical grade acaricides: cypermethrin at 923.8 mg/mL; chlorpyrifos at 974 mg/mL; ivermectin at 970.8 mg/g (Sigma Chemical Co., St Louis, MO, USA); and fipronil at 960.6 mg/g (BASF Chemicals®, Paulínia, São Paulo, Brazil). For amitraz, a commercial formulation at 125 mg/mL (Triatox®, MSD Saúde Animal, São Paulo, Brazil) was employed. A Larval Packet Test (LPT) was performed for cypermethrin and chlorpyrifos, and a modified LPT for amitraz (Stone & Haydock, 1962; Miller et al., 2002). A Larval Immersion Test (LIT) was performed for fipronil and ivermectin due to their higher sensitivity for diagnosing tick resistance to those compounds (Klafke et al., 2012; Castro Janer et al., 2015).

Stock solutions and dilutions of the five evaluated acaricides were prepared. For cypermethrin, 216.5 mg were added to 20 mL of a trichloroethylene (TCE) + olive oil solution to achieve a concentration of 10 mg/mL, and serial dilutions of 8, 6, 4, 3, 2, 1, 0.8, 0.4, 0.2, and 0.1 mg/mL were prepared. For chlorpyrifos, 770 mg were added to 15 mL of a TCE + olive oil solution to achieve a concentration of 40 mg/mL, and serial dilutions of 30, 20, 10, 8, 6, 3.3, 0.6, 0.4, 0.3, 0.2, 0.1, 0.06, and 0.03 mg/mL were prepared. For amitraz, 30 mg diluted in 0.24 mL were added to 2.76 mL of a TCE + olive oil solution to achieve a concentration of 10 mg/mL, then serial dilutions of 1, 0.8, 0.6, 0.4, 0.2, 0.1, 0.06, 0.02, and 0.004 mg/mL were prepared. For ivermectin, 103 mg was added to 10 mL of pure to achieve a concentration of 10 mg/mL, and serial dilutions of 100, 50, 25, 12.5, 6.25, 3.125, 1.56, and 0.78 mg/L were prepared in an acetone 100 mg/mL + Triton 4 mg/mL solution. For fipronil, 104.1 mg was diluted in a solution of acetone 10 mg/mL + Triton 0.4 mg/mL until a concentration of 10 mg/L was achieved, then serial dilutions of 8, 5, 4, 2.5, 2, 1.5, 1.2, 1, 0.8, 0.6, and 0.4 mg/L were prepared in an acetone 100 mg/mL + Triton 4 mg/mL solution. No active ingredient was added to the control solutions.

For each acaricide dilution of cypermethrin, chlorpyrifos, and amitraz, filter papers (7.5 cm × 8.5 mm, Whatman No. 1, Whatman Inc., Maidstone, England) were impregnated with 0.67 mL. A cluster of approximately 100 (range: 80–120) unfed and vigorous larvae, aged 14-21 days, was transferred to each paper and sealed in a packet. Three replications were made for each dilution of the five acaricides and the negative control group. The packets were incubated in a BOD chamber at 27°C to 28°C and 80% to 90% relative humidity for 24h. After incubation, the numbers of dead and live larvae were recorded. If the control group mortality exceeded 10%, the tick strain was discarded.

For the LIT, stock solutions of ivermectin and fipronil were prepared. For each acaricide dilution, 1.5 mL Eppendorf tubes were filled with 0.5 mL, and approximately 400 to 500 unfed larvae between 14 and 21 days old were submerged in each tube and in the control group for 10 minutes. Then, the liquid was discarded, and 100 larvae were transferred to filter papers (7.5 cm × 8.5 mm, Whatman No. 1), forming a sealed packet. The packets were incubated in a BOD chamber at 27°C to 28°C and 80% to 90% relative humidity for 24h. Three replications were made for each dilution. At the end of incubation, the total number of dead and live larvae was determined. For each test, if the control group mortality exceeded 10%, the tick strain was discarded.

To obtain a dose-response curve covering a mortality range from 5% to 95% and to ensure adequate discrimination between susceptible and resistant populations, the dilutions of the acaricides were adjusted according to preliminary assays and reference protocols described for *R. microplus* (Stone & Haydock, 1962; Miller et al., 2002; Klafke et al., 2012; Castro Janer et al., 2015; FAO, 2025). The highest concentrations were chosen to guarantee near-total mortality in susceptible populations, while the lowest concentrations allowed detection of shifts in susceptibility. When necessary, concentration ranges were adjusted according to the expected susceptibility of field populations.

### Statistical analysis

The median lethal concentration (LD_50_) was determined for each acaricide using a probit analysis of mortality. The analysis was performed in Polo-Plus software (LeOra Software, 2004. PoloPlus. Probit and Logit Analysis, Berkeley, CA, USA). All the LD_50_ values of the 14 field tick strains obtained were compared with the LD_50_ values of POA strain (Reck et al., 2014) to obtain a resistance ratio (RR_50_). A tick strain was considered resistant when RR_50_ was > 2.0.

It was determined whether the independent variables derived from the questionnaire influenced the RR_50_ distribution. Because the RR_50_ variable is continuous, the RR_50_ medians of both categories were compared for each question using the nonparametric Mann-Whitney U test. Statistical analyses were performed using Jamovi version 2.6 (Love et al., 2025). Graphs were generated using GraphPad Prism version 8.0.2 for Windows (Motulsky, 2019).

## Results

### Farm features

On the sampled farms, the average number of adult cattle was 51 (9-145 animals), and the average area was 57 ha. All farms produced cattle in extensive systems, had animals infested with *R. microplus*, and implemented acaricide control programs. According to the questionnaire, most farms were dedicated to dairy production (57.14%, 8/14), while the rest, to beef production (42.85%, 6/14). Most of the herds were composed of *Bos indicus* cattle (57.14%, 8/14), followed by farms with *Bos taurus* and crossbred cattle (42.85%, 6/14). Concerning cattle purchases, five farmers (35.71%, 5/14) purchased cattle from other farms located in regions where climatic conditions hinder tick development, such as the Caatinga biome, and two (14.28%, 2/14) reported purchasing cattle from regions where climatic conditions favor the parasite, such as the Atlantic Forest biome of the State of Bahia (unpublished results). The other seven farmers did not introduce cattle from other farms.

Regarding *R. microplus* infestation, some farmers reported the tick was present seasonally (35.71%, 5/14), while others reported it occurred year-round (28.57%, 4/14). Five farmers did not answer this question. Concerning acaricide treatments, some farmers reported treating only when high tick infestation was observed in cattle (42.85%, 6/14), while others treated systematically or prophylactically (35.71%, 5/14). Three farmers did not answer this question. Regarding the choice of acaricide compounds, half of the farmers chose based on a veterinary recommendation (50%, 7/14), whereas others were based on their own criteria (28.57%, 4/14). Three farmers did not answer this question. Regarding the frequency of treatments, most farmers performed >2 treatments per year (57.14%, 8/14), while others performed ≤2 treatments per year (21.42%, 3/14). Three farmers did not answer this question. Regarding the acaricide application method, farmers performed injectable (71.43%, 10/14), pour-on (57.14%, 8/14), and/or spray formulations (57.14%, 8/14). Half of the farmers did not alternate the acaricide compounds (50%, 7/14). A group of farmers used multiple acaricide classes simultaneously (35.71%, 5/14), and some reported using different products within the same chemical class (35.71%, 5/14). Regarding the acaricide compound, most farmers reported using avermectins (78.57%, 11/14), a mixture of cypermethrin and chlorpyrifos (71.43%, 10/14), and a small group reported using fipronil (21.42%, 3/14). No one reported using amitraz or other acaricides not included in this report (fluazuron or fluralaner).

### Acaricide resistance bioassay

A total of 14 cypermethrin, 11 chlorpyrifos, 14 amitraz, 12 ivermectin, and 14 fipronil tests were performed. In five farms, it was not possible to run bioassays for all five acaricides due to logistical and biological constraints, specifically insufficient larval numbers, or extreme resistance levels (limited reliable probit analysis). In those cases, tick resampling was unfeasible, as it would have required farmers to suspend acaricide treatments again, making further testing operationally and economically impractical.

### Frequency of resistance

**Figure 2:**
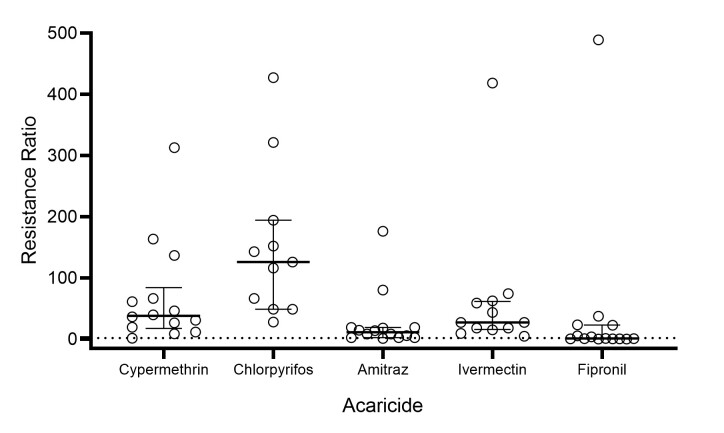
Distribution of resistance ratios (RR_50_) of *Rhipicephalus microplus* populations from farms in the Recôncavo baiano region, State of Bahia, Brazil. The results are presented as medians (horizontal lines) and interquartile ranges (error bars). The dashed line indicates the RR_50_ threshold (2.0) from which ticks would be considered resistant. Circles represent farms (cypermethrin *n*= 14; chlorpyrifos *n*= 11; amitraz *n*= 14; ivermectin *n*= 12; fipronil *n*= 14).

Resistance to cypermethrin was observed in 92.86% of the sampled farms (13/14); for chlorpyrifos, in 100% (11/11); for amitraz, in 92.86% (13/14); for ivermectin, in 100% (12/12); and for fipronil, in 42.86% (6/14) (Figure 2).

### Multi-resistance profiles

Tick strains resistant to three or more acaricides (multi-resistant strains) were found in all 14 evaluated farms. Resistance to three compounds was observed in 14.28% (2/14) of farms, one showed resistance to chlorpyrifos, amitraz, and ivermectin, and another to cypermethrin, chlorpyrifos, and ivermectin. Resistance to four compounds occurred in 78.57% (11/14) of the farms. The most frequent resistance profile involved cypermethrin, chlorpyrifos, amitraz, and ivermectin (42.56%, 6/14), followed by cypermethrin, amitraz, ivermectin, and fipronil (21.43%, 3/14) and by cypermethrin, chlorpyrifos, amitraz, and fipronil (14.29%, 2/14). Resistance to the five compounds was observed only in the tick strain from a farm in Santo Antônio de Jesus (7.14%, 1/14) (Tables 1 [Table t02] [Table t03] [Table t04]-5).

**Table 1:** Classification of cypermethrin resistance status of *Rhipicephalus microplus* populations from the Recôncavo baiano region, Brazil, based on resistance ratios (RR_50_).

**Strain**		**LD_50_**	**CI95% LD_50_**	**RR_50_**	**Status**
	POA	150.66			
1	Santo Amaro da Purificação 1	1733.44	1187.83-2678.61	11.51	R
2	Santo Amaro da Purificação 2	5478.5	3408.8-14274	36.36	R
3	Santo Amaro da Purificação 3	6980.3	5783.9-8927.5	46.33	R
4	Santo Amaro da Purificação 4	2945.9	2223.95-4201.19	19.55	R
5	Santo Amaro da Purificação 5	4036.9	2974.48-6146.72	26.79	R
6	Conceição do Almeida	4666.54	4040.08-5549.16	30.97	R
7	São Felipe	220.31	162.02-283.63	1.46	S
8	Cruz das Almas	1325.04	1067.63-1901.66	8.79	R
9	Sapeaçú	24673	15273-183110	163.77	R
10	Cabaceiras do Paraguaçú	20632	13429-114430	136.94	R
11	Dom Macedo Costa	5988.36	5358.53-6715.19	39.75	R
12	Santo Antônio de Jesus	47177	24818-138960	313.14	R
13	Muritiba	9244.8	7620.9-12465	61.36	R
14	São Gonçalo dos Campos	10052	7615.2-14617	66.72	R

Note: LD, lethal dose. CI, confidence interval. RR, resistance ratio. R, resistant. S, susceptible. POA, Porto Alegre susceptible reference strain.

**Table 2 t02:** Classification of chlorpyrifos resistance status of *Rhipicephalus microplus* populations from the Recôncavo baiano region, Brazil, based on resistance ratios (RR_50_).

**Strain**		**LD_50_**	**CI95% LD_50_**	**RR_50_**	**Status**
	POA	85.21			
1	Santo Amaro da Purificação 1	5661.3	2929.7-12413	66.44	R
2	Santo Amaro da Purificação 2	-	-	-	-
3	Santo Amaro da Purificação 3	12185	9875.1-15407	143.00	R
4	Santo Amaro da Purificação 4	9907.7	7364.1-13814	116.27	R
5	Santo Amaro da Purificação 5	27407	20189-40430	321.64	R
6	Conceição do Almeida	-	-	-	N.a.
7	São Felipe	4178.16	3367.67-5106.97	49.03	R
8	Cruz das Almas	36432	25317-60265	427.55	R
9	Sapeaçú	-	-	-	N.a.
10	Cabaceiras do Paraguaçú	4163.9	3190.67-5221.48	48.87	R
11	Dom Macedo Costa	16564	11843-24328	194.39	R
12	Santo Antônio de Jesus	10738	8435.7-13230	126.02	R
13	Muritiba	2392.8	1026.2-7830.3	28.08	R
14	São Gonçalo dos Campos	12960	10649-15680	152.09	R

Note: LD, lethal dose. CI, confidence interval. RR, resistance ratio. R, resistant. S, susceptible. N.a., not applicable. POA, Porto Alegre susceptible reference strain. Only 12 farms were successfully tested for this acaricide.

**Table 3 t03:** Classification of amitraz resistance status of *Rhipicephalus microplus* populations from the Recôncavo baiano region, Brazil, based on resistance ratios (RR_50_).

**Strain**		**LD_50_**	**CI95% LD_50_**	**RR_50_**	**Status**
	POA	55.61			
1	Santo Amaro da Purificação 1	146.56	126.06-169.37	2.64	R
2	Santo Amaro da Purificação 2	321.93	249.48-424.78	5.79	R
3	Santo Amaro da Purificação 3	146.3	116.75-184.13	2.63	R
4	Santo Amaro da Purificação 4	141.03	81.98-261.82	2.54	R
5	Santo Amaro da Purificação 5	784.14	597.92-1142.07	14.10	R
6	Conceição do Almeida	458.62	210.95-1891.07	8.25	R
7	São Felipe	470.51	384.43-584.21	8.46	R
8	Cruz das Almas	1008.58	652.29-1955.62	18.14	R
9	Sapeaçú	1073.72	987.14-1291.83	19.31	R
10	Cabaceiras do Paraguaçú	818.96	503.12-1815.74	14.73	R
11	Dom Macedo Costa	60.84	38.08-80.7	1.09	S
12	Santo Antônio de Jesus	9813.3	2401.4-585200	176.48	R
13	Muritiba	4471.8	1288.6-167020	80.42	R
14	São Gonçalo dos Campos	1071.67	980.23-1235.48	19.27	R

Note: LD, lethal dose. CI, confidence interval. RR, resistance ratio. R, resistant. S, susceptible. POA, Porto Alegre susceptible reference strain.

**Table 4 t04:** Classification of ivermectin resistance status of *Rhipicephalus microplus* populations from the Recôncavo baiano region, Brazil, based on resistance ratios (RR_50_).

**Strain**		**LD_50_**	**CI95% LD_50_**	**RR_50_**	**Status**
	POA	2.67			
1	Santo Amaro da Purificação 1	73.05	52.23-118.96	27.32	R
2	Santo Amaro da Purificação 2	48.65	1.66-8.35	18.20	R
3	Santo Amaro da Purificação 3	48.66	30.46-81.23	18.20	R
4	Santo Amaro da Purificação 4	40.68	35.36-47.46	15.21	R
5	Santo Amaro da Purificação 5	12.69	7.95-19.96	4.74	R
6	Conceição do Almeida	24.67	17.11-40.44	9.23	R
7	São Felipe	158.29	113.13-280.12	59.20	R
8	Cruz das Almas	73.19	60.41-92.49	27.37	R
9	Sapeaçú	199.33	100.66-1182.15	74.54	R
10	Cabaceiras do Paraguaçú	-	-	-	N.a.
11	Dom Macedo Costa	116.76	94.27-156.79	43.67	R
12	Santo Antônio de Jesus	1119.9	397.06-27525	418.81	R
13	Muritiba	167.7	119.09-545.07	62.72	R
14	São Gonçalo dos Campos	-	-	-	N.a.

Note: LD, lethal dose. CI, confidence interval. RR, resistance ratio. R, resistant. S, susceptible. N.a., not applicable. POA, Porto Alegre susceptible reference strain. Only 12 farms were successfully tested for this acaricide.

**Table 5:** Classification of fipronil resistance status of *Rhipicephalus microplus* populations from the Recôncavo baiano region, Brazil, based on resistance ratios (RR_50_).

**Strain**		**LD_50_**	**CI95% LD_50_**	**RR_50_**	**Status**
	POA	1.09			
1	Santo Amaro da Purificação 1	0.1	0.03-0.19	0.09	S
2	Santo Amaro da Purificação 2	4.28	0.34-0.49	3.93	R
3	Santo Amaro da Purificação 3	0.56	0.52-0.6	0.51	S
4	Santo Amaro da Purificação 4	0.51	0.38-0.63	0.47	S
5	Santo Amaro da Purificação 5	0.35	0.21-0.46	0.32	S
6	Conceição do Almeida	25.5	13.4-107.1	23.35	R
7	São Felipe	0.78	0.73-0.84	0.72	S
8	Cruz das Almas	0.97	0.91-1.03	0.89	S
9	Sapeaçú	534.5	64.26-	489.47	R
10	Cabaceiras do Paraguaçú	41.02	21.04-137.95	37.56	R
11	Dom Macedo Costa	1.36	1.02-1.77	1.24	S
12	Santo Antônio de Jesus	5.77	3.7-13.28	5.29	R
13	Muritiba	0.53	0.48-0.57	0.48	S
14	São Gonçalo dos Campos	24.91	9.73-425.98	22.81	R

Note: LD, lethal dose. CI, confidence interval. RR, resistance ratio. R, resistant. S, susceptible. POA, Porto Alegre susceptible reference strain.

### Intensity of resistance

The median RR_50_ values were 38.06 (interquartile range [IQR]: 17.54; 84.28) for cypermethrin, 126 (IQR: 49.03; 194.4) for chlorpyrifos, 11.28 (IQR: 2.64;19.28) for amitraz, 27.35 (IQR: 15.96;61.84) for ivermectin, and 1.06 (IQR: 0.49;22.95) for fipronil (Figure 2). The highest RR_50_ values for cypermethrin, amitraz, and ivermectin were observed in the tick strain from a farm in Santo Antônio de Jesus. In contrast, the highest values for chlorpyrifos were recorded in the tick strain from a farm in Cruz das Almas, and for fipronil in the tick strain from a farm in Sapeaçú (Tables 1 [Table t02] [Table t03] [Table t04]-5).

### Variable associations

Regarding the comparison between RR_50_ medians of both categories for each independent variable from the questionnaire, the RR_50_ median of amitraz and fipronil was higher (*P* < 0.05) in farms with *Bos taurus* and crossbred cattle than those with *Bos indicus* cattle, and higher (*P* < 0.05) in farms destined to dairy production than those destined to beef production. The RR_50_ median for fipronil was higher (*P* < 0.05) in farms that chose the acaricide based on veterinary recommendations without a resistance test than in those that chose based on their own criteria (Table 6).

**Table 6:** Comparison of median resistance ratios (RR50) for five acaricides across categories of 13 independent variables from 14 farms in the Recôncavo baiano region, Brazil.

**Independent variable** **Category**	**Cypermethrin**			**Chlorpyrifos**			**Amitraz**			**Ivermectin**			**Fipronil**		
	**Median (RR_50_)**	**IQR (RR_50_)**	***p***	**Median (RR_50_)**	**IQR (RR_50_)**	***p***	**Median (RR_50_)**	**IQR (RR_50_)**	***p***	**Median (RR_50_)**	**IQR (RR_50_)**	***p***	**Median (RR_50_)**	**IQR (RR_50_)**	***p***
**Genetic composition**			0.282			1			**0.04**			0.272			**0.043**
*Bos indicus*	31.57	17.54-41.39		121.14	62.09-155.85		4.21	2.61-9.87		22.76	17.45-47.55		0.66	0.43-1.91	
*Bostaurus* and crossbred cattle	64.04	38.57-119.38		100.48	43.67-220.95		18.71	15.58-19.3		62.72	27.37-74.54		23.08	6.37-34.01	
**Production system**			0.059			0.48			**0.008**			0.153			**0.008**
Beef	23.2	13.5-36.5		130	78.9-182		2.63	2.56-7.01		22.8	16-39.6		0.49	0.36-0.67	
Dairy	64	35-144		87.4	33.3-146		18.7	13.1-34.6		62.7	22.8-247		14	3.17-26.9	
**Cattle origin**			0.57			0.8			0.86			0.38			0.86
From the Caatinga biome	46.33	39.75-163.77		134.51	111.13-155.85		2.64	2.63-19.31		43.67	27.32-74.54		1.24	0.51-5.29	
From the Atlantic Forest biome	34.09	17.76-50.41		100.56	74.8-126.32		13.87	11.16-16.57		751.27	405.24-1097.3		11.76	6.24-17.29	
**Tick infestation occurrence**			0.29			0.34			0.19			0.34			0.91
All through the year	38.05	29.47-46.49		173.24	114.07-252.68		11.96	4.62-18.42		35.52	25.08-393.59		2.58	1.15-8.65	
Part of the year	136.94	61.36-163.77		48.95	43.67-68.28		19.31	14.73-80.42		68.63	61.84-160.61		5.29	0.72-37.56	
**Criteria for acaricide treatments**			0.93			0.61			0.43			0.52			0.93
Systematic or prophylactic	46.33	11.54-136.94		57.73	48.99-85.58		8.46	2.64-14.73		43.26	25.04-63.04		0.72	0.51-37.56	
High tick infestation in cattle	50.55	37.21-65.38		139.05	52.56-183.81		18.71	8.88-65.13		53.19	31.45-329.79		2.58	0.98-4.95	
**Acaricide purchase**			0.23			0.39			0.51			0.45			**0.006**
Personal election	28.92	8.99-50.09		57.73	43.79-85.58		5.55	2.64-26.45		43.26	25.04-60.08		0.49	0.38-0.56	
Veterinary recommendation without a resistance test	66.72	38.05-150.35		139.05	68.16-183.81		18.14	10.26-19.29		59.1	31.44-332.74		5.29	2.58-30.18	
**Frequency of acaricide treatments**			0.92			0.67			0.92			0.49			1
≤2 treatments per year	39.75	20.60-176.44		126.02	87.52-160.2		8.46	4.77-92.47		59.2	51.43-239		1.24	0.98-3.27	
>2 treatments per year	53.84	30.15-84.28		66.44	38.47-147.55		16.44	5-19.28		27.37	22.76-68.63		2.41	0.5-26.5	
**Injectable acaricide formulations**			0.91			0.4			0.36			0.73			0.36
No	61.36	61.36-61.36		28.08	28.08-28.08		80.42	80.42-80.42		62.72	62.72-62.72		0.48	0.48-0.48	
Yes	43.04	17.72-119.38		126.02	49.03-152.09		11.59	3.43-18.99		43.67	27.32-74.54		2.58	0.76-18.43	
**Pour-on acaricide formulations**			0.76			0.83			0.76			0.57			0.28
No	46.33	28.92-179.73		126.02	96.23-134.51		2.64	2.63-89.56		27.32	22.76-223.06		0.51	0.3-2.9	
Yes	50.55	29.47-84.28		49.03	38.47-173.24		16.43	7.79-19.28		59.2	35.52-68.63		2.58	0.85-26.5	
**Spray acaricide formulations**			0.28			1			0.76			0.65			0.5
No	11.51	6.48-39.1		66.44	57.73-109.3		8.46	5.55-13.9		59.2	43.26-751.3		0.72	0.4-11.8	
Yes	53.84	38.9-143.6		126.02	38.47-168.7		16.43	5-34.6		43.67	22.78-68.6		2.58	0.79-13.4	
**Rotation of an acaricide compound**			0.79			0.17			0.16			0.21			1
No	61.36	23.93-115.2		57.73	33.32-111.1		19.27	7.12-49.9		62.72	43.26-246.7		3.93	0.6-14	
Yes	43.04	32.01-69		168.69	119.47-2527		8.68	2.24-15.6		27.37	22.78-35.5		1.06	0.79-10.3	
**Multiple acaricide classes used simultaneously**			0.73			0.89			0.33			1			0.44
No	46.33	11.51-66.72		96.23	43.79-145.27		18.14	5.79-19.31		59.2	27.32-74.54		0.89	0.51-5.29	
Yes	88.34	64.05-112.64		121.63	85.25-158.01		7.91	4.5-11.32		43.67	43.67-43.67		19.4	10.32-28.48	
**Use different products with the same compound**			0.66			0.69			0.79			0.39			1
No	56.52	20.21-139.51		126.02	66.44-143		13.87	4.09-19.3		66.87	35.29-332.74		3	0.56-18.43	
Yes	39.75	36.36-61.36		48.87	28.08-194.39		14.73	5.79-18.14		35.52	25.08-48.43		1.24	0.89-3.93	

Note: Mann-Whitney U test. Variables with *P* values in bold were considered significant. RR, resistance ratio. Interquartile range, IQR.

## Discussion

This is the first research to diagnose high intensity of resistance (RR_50_) to cypermethrin, chlorpyrifos, and ivermectin in *R. microplus* larvae from cattle farms in the State of Bahia, Brazil, using standardized larval bioassays and based on a susceptible reference strain. Amitraz and fipronil had the lowest RR_50_ median.

The RR_50_ medians of four acaricides evaluated in the current study were above the susceptibility threshold, and the resistance situation for cypermethrin, chlorpyrifos, and ivermectin in farms in the Recôncavo baiano region was the most critical. Although ivermectin was the compound most effective against cattle tick in studies performed with Adult Immersion Test (AIT) in farms from the Caatinga biome of the State of Bahia (Raynal et al., 2013), we found a high level of resistance to this compound, even showing a higher median intensity of resistance than other studies performed with LIT in Brazil (Klafke et al., 2006; Vilela et al., 2020). The high level of resistance to this compound observed in our research may be related to its frequent use, as avermectins were the chemical group most used in the sampled farms, followed by a mixture of cypermethrin and chlorpyrifos. This mixture was mainly applied in spray, an administration method that can facilitate its misuse and, consequently, favor the resistance (Spickett & Fivaz, 1992). It seems that the intensity of resistance to cypermethrin and chlorpyrifos has been increasing over the years, as our findings exceed levels reported in other studies using LPT in Brazil (Mendes et al., 2011; Nogueira Domingues et al., 2012; Vilela et al., 2020). Because the smaller sample size likely influenced the higher chlorpyrifos RR_50_ variance (11 farms), the median RR_50_ of this compound should be interpreted with caution.

In studies performed in the Atlantic Forest biome, fipronil has been the most effective acaricide (Campos Júnior & Oliveira, 2005; Spagnol et al., 2010; Raynal et al., 2018). Even though those investigations have been limited to AIT, they are consistent with our finding that fipronil has the lowest level of resistance in the Recôncavo baiano. Although fipronil was the only compound with a median RR_50_ below the threshold for resistance and the median RR_50_ was higher than those reported in similar studies in Northeastern Brazil (Vilela et al., 2020), 42.86% of the farms evaluated were currently classified as resistant to this compound. Regarding amitraz, although its use was not reported, we found a higher level of resistance than in other regions of the Brazilian Northeast (Vilela et al., 2020).

The resistance intensity to amitraz and fipronil was higher in dairy than in beef cattle systems, although all farmers stated that amitraz had never been applied. A similar pattern was observed for fipronil, which showed resistance in four dairy farms that reported not using it (unshown data). This situation may be due to the movement of cattle or other animals (goats, sheep, donkeys, horses, or wild animals) that could introduce resistant ticks into farms (Miraballes et al., 2019). In this region of Northeast Brazil, and likely in other regions of Brazil with numerous small farms, the spread of acaricide resistance appears to be largely driven by migration, an important factor in the emergence of resistance on a farm (Rodriguez-Vivas et al., 2018). Overall, fipronil and amitraz had the lowest median RR_50_ values.

Regarding the variable association analysis, the higher resistance levels observed in dairy farms (comprising *Bos taurus* and crossbred cattle) may be due to the greater tick infestation susceptibility in these breeds, leading to increased treatment frequency. Acaricidal resistance has been associated with dairy farms, likely because *Bos taurus* cattle require more acaricide treatments than *Bos indicus* cattle (Jonsson, 2006; Pérez-Otáñez et al., 2024). In contrast with results from other regions of Brazil, where the highest levels of multiple resistance were associated with larger herds (Ferreira et al., 2025), in the Recôncavo baiano, we observed higher resistance in smaller farms. A similar situation was observed in Mexico, where the highest levels of resistance occurred in small farms with <50 animals (Fernández-Salas et al., 2012). The associations found in this research should be interpreted with caution due to the small number of farms, the lack of adjustment for multiple testing, which increases the risk of type I error (false-positive findings), and the fact that the associations relied on owners’ perceptions, which may vary over time. More robust analyses are necessary.

The questionnaire results reveal that producers in Recôncavo baiano lacked a plan for tick infestation control, which favors the increase in the tick population and the number of sequential treatments. The most appropriate protocol would be strategic control, where all animals are treated simultaneously, interrupting and synchronizing the tick’s biological cycle. In this way, tick infestation is kept under control, reducing the number of acaricide applications, costs, contamination, and delaying the occurrence of resistance (Nicaretta et al., 2023). The first treatment should be performed at the beginning of the summer, when the larval density in the pasture is low, and the re-treatments when ticks < 4 mm are observed in more than 30% of the herd (Nicaretta et al., 2021).

To determine the still-effective acaricides to include in the strategic control protocol in the Recôncavo baiano region, as in other regions, each cattle farmer should assess the resistance status on their own property before implementing a treatment strategy. To assess the efficiency of commercial compounds, field studies should be performed comparing infestation levels in bovines (20 treated and five controls) at baseline and at seven and 21 days after treatment (Holdsworth et al., 2022). If it is not viable to perform these studies, patronized larval bioassays are a practical option (FAO, 2025). Although the direct extrapolation of *in vitro* results to *in vivo* situations may not always be accurate (Klafke et al., 2024), quantitative analyses, such as LPT and LIT allow the determination of acaricide resistance levels (Ferreira et al., 2025). Although fipronil and amitraz resistance were frequent in our research, because of their lower intensity of resistance, both compounds should be included in the formulation of strategic control protocols. Because of the need for various compounds without resistance, fluazuron would have to be included in resistance test protocols, since it was not included in our research, and there are reports of a high degree of resistance in other regions (Ferreira et al., 2025), as for fluralaner, for which there are no reports of resistance.

Although we had to choose properties interested in participating by convenience (non-randomized sampling), our results could reflect the reality of the Recôncavo baiano and represent a contribution to the understanding of the acaricide resistance in regions and farms with characteristics very similar to our sample (small and medium properties located in tropical regions from the Atlantic forest near the Caatinga biome).

## Conclusions

Our results revealed a high intensity of resistance to the main acaricides used for *Rhipicephalus microplus* control in farms in the Recôncavo baiano region. Multi-resistant tick populations were detected across all farms studied, and several properties exhibited high levels of resistance to acaricides that had reportedly never been used. Since amitraz and fipronil showed the lowest average resistance levels, formulating strategic control protocols based on these compounds is recommended.

## Data Availability

All data generated in this study are included in this published article.
